# Endoscopic Double-Pigtail Catheter (EDPC) Internal Drainage as First-Line Treatment of Gastric Leak: A Case Series during Laparoscopic Sleeve Gastrectomy Learning Curve for Morbid Obesity

**DOI:** 10.1155/2020/8250904

**Published:** 2020-12-23

**Authors:** Gianni Lazzarin, Marino Di Furia, Lucia Romano, Alessandra Di Sibio, Carla Di Giacomo, Loreto Lombardi, Antonio Giuliani, Mario Schietroma, Beatrice Pessia, Francesco Carlei, Michele Marchese

**Affiliations:** ^1^Department of Biotechnological and Applied Clinical Sciences, ASL1 Abruzzo, San Salvatore Hospital, L'Aquila, Italy; ^2^Department of Radiology, ASL1 Abruzzo, San Salvatore Hospital, L'Aquila, Italy; ^3^Surgical Endoscopy Unit, ASL1 Abruzzo, San Salvatore Hospital, L'Aquila, Italy

## Abstract

**Objectives:**

The prevalence of morbid obesity has dramatically increased over the last several decades worldwide, currently reaching epidemic proportions. Gastric leak (GL) remains the potentially fatal main complication after sleeve gastrectomy (SG) for morbid obesity. To our knowledge, there are no standardized guidelines for GL treatment after laparoscopic sleeve gastrectomy (LSG) yet. The aim of this study was to represent our institutional preliminary experience using the endoscopic double-pigtail catheter (EDPC) as the method of internal drainage and propose it as first-line treatment in case of GL after LSG.

**Methods:**

One hundred and seventeen patients were admitted to our surgical department and underwent laparoscopic sleeve gastrectomy (LSG) for morbid obesity from March 2014 to June 2019. In 5 patients (4.3%) of our series, GL occurred as a complication of LSG. EPDC was the stand-alone procedure of internal drainage and GL first-line treatment. The internal pig tail was endoscopically removed from 30^th^ to 40^th^ POD in all cases.

**Results:**

Present data (clinical, biochemical, and instrumental tests) showed a complete resolution of GL, with promotion of a pseudodiverticula and complete re-epithelialization of leak. Follow-up was more strict than usual (clinical visit and biochemical test on 7^th^, 14^th^, and 21^st^ day after discharge; a CT scan with gastrografin on 30^th^ day from discharge if clinical visit and exams were normal).

**Conclusion:**

This was a preliminary retrospective observational study, conducted on 5 patients affected by GL as a complication of LSG for morbid obesity. EDPC maintains the safety, efficacy, and nonexpensive characteristic and may be proposed as better first-line treatment in case of GL after bariatric surgery.

## 1. Introduction

The prevalence of morbid obesity has dramatically increased over the last several decades worldwide, currently reaching epidemic proportions. The total number of bariatric surgical procedures recorded in 2018 by the “International federation for the surgery of obesity” was 394.431 [1]. The most performed procedures were Roux-en-Y gastric bypass (RYGB) 41.9%, sleeve gastrectomy (SG) 32.6%, gastric banding (GB) 12%, and one-anastomosis gastric bypass procedures (MINI bypass) 5%. The global trends from 2014 to 2018 seem to show a decrease in RYGB in favor of SG (38.3% vs. 45.9%) [1] that will probably represent the common bariatric surgery procedure in a not faraway future.

Gastric leak remains the potentially fatal main complication after SG. To our knowledge, there are no standardized guidelines for its treatment [2].

The aim of this study is to represent good results using the endoscopic double-pigtail catheter (EDPC) as first-line treatment in case of gastric leak (GL) after laparoscopic SG (LSG). We report our institutional experience.

Gastric leak (GL) can be defined as the leak of luminal contents from a surgical joint between two hollow viscera (or between viscera and the peritoneal cavity) and is classified based on the onset timing of GL as early (≤3 days after surgery), intermediate (≥4 and ≤7 days after surgery), and late (>8 days after surgery) [3].

GL is certainly the most common and life-threatening complication of laparoscopic SG (LSG), with an incidence of 1–10% of patients in published series [4]. Its incidence can rise to 16–20% following repeated operative surgery [4]. Therefore, surgical redo cannot be considered the first-line treatment in case of GL.

Others major complications are abscess (that usually result from gastric fistulas and, in many cases, can be drained percutaneously under image guidance) and hemorrhage (that can be often treated by percutaneous embolization or medical sustain).

Classically, leaks tend to appear between 5 and 6 postoperative days because of a lack of staple line integrity [3, 5, 6]. The typical location of GL is the proximal third of the stomach, close to the gastroesophageal junction (near the angle of His- 85.7%); it less commonly occurs in the distal third (14.3%) [4]. We would like to underline that GL management is still relatively “empiric,” without standardized guidelines yet [3].

Endoscopic double-pigtail catheter (EDPC), as a method of internal drainage, plays an important role in the minimally invasive management of various postoperative bariatric surgical complications [7].

### 1.1. Case Reports

All patients of our series underwent laparoscopic sleeve gastrectomy for obesity at the Unit of General Surgery, University of L'Aquila.

Complicated patients (Table 1) were retrospectively chosen by the authors or their colleagues. In these patients, GL was located in all cases near the angle of His. The global median age of our series (Table 2) was 45 years (18–67 age range), while the median age of GL group was 44.4 years. The mean BMI at surgery time was 41.6 Kg/m^2 (36.8–45 Kg/m^2 BMI range). The patients complicated had no previous bariatric procedures (such as endoscopic placement of B.I.B.) or bariatric surgery (Table 1).

### 1.2. Case 1

**Figure 1:**
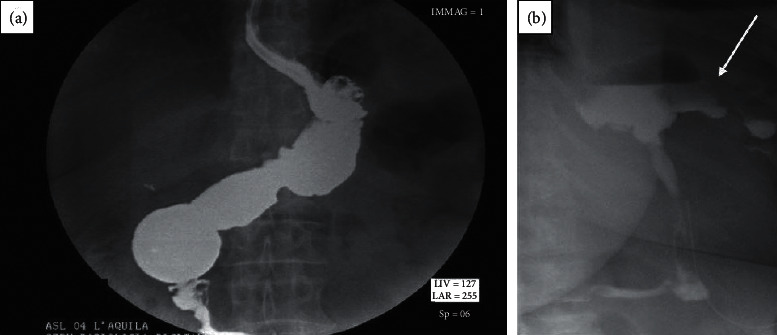
Gastrografin swallow test (GST). (a) Normal 2^nd^ POD GST: tightness of the linear suture is confirmed, and presence of gastric leak is excluded. (b) 2^nd^ POD GST of our early complicated patient: it clearly shows the gastric leak (white arrow) close to the gastroesophageal junction near the angle of His.

**Figure 2:**
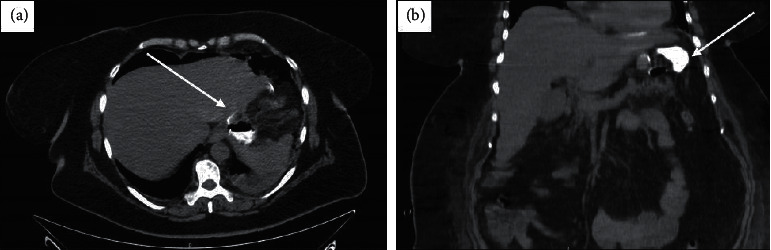
The early complicated patient, shown on axial (a) and coronal (b) CT-scan during the portal phase. CT images show the GL and corresponding collection on the 2^nd^ POD (Figure 2(a), white arrow). The gastric fistula linking the stomach and enhanced collection (white arrow) are shown.

**Figure 3:**
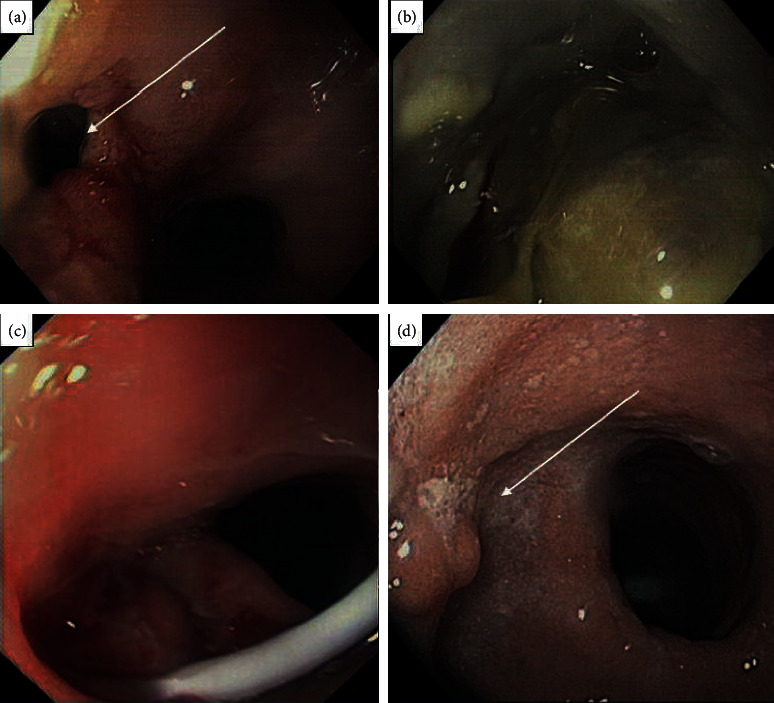
Endoscopic views of an early complicated patient. The GL documented with GST and CT scans now appears evident at endoscopic inspection ((a)) (with arrow). A large walled-off perifistular collection suspected at GST (Figure 1(b)) is confirmed at the endoscopic inspection (b). (c) EDPC positioned. (d) Endoscopic control at the 28^th^ POD: internal drainage acts like a foreign body and promotes the physiological re-epithelization of the leak (white arrow).

The clinical scenario of patient 1 was suggestive of early GL and abdominal abscess, with tachycardia (above 120 beat/min), white blood cell count was 18.5 × 10^9^/L with 78.9% neutrophils, CRP was 22 mg/dL, and PCT was 15 ng/ml, with fever and abdominal pain on the 1^st^ POD. Surgical drainage output appeared corpuscular. GST (2^nd^ POD) revealed GL of gastroesophageal junction (Figure 1(b), further investigated with contrast-enhanced CT scan (CT–Figures 2(a) and 2(b); 3^rd^ POD) and endoscopic control (Figure 3(a)). All clinical and instrumental findings supported diagnosis of GL. EDPC was left in place on the 4^th^ POD.

### 1.3. Case 2

Patient 2 corresponded to a young female affected by morbid obesity. The postoperative course after LSG was regular, and the patient was completely asymptomatic at the beginning: biochemical tests of the 1^st^ and 3^rd^ POD did not reveal abnormalities; even GST on the 2^nd^ postop day was negative. Diagnosis was suggested by clinical abnormalities appearing on 4^th^-5^th^ POD (tachycardia, fever, dyspnea, and sudden increase of CRP) and confirmed by a CT scan with gastrografin per os. EDPC was placed on the 7^th^ POD.

### 1.4. Case 3

A female patient of suffered an intermediate GL occurred on the 8^th^ POD. Also, in this case, diagnosis was suggested by clinical abnormalities appeared on 6^th^-7^th^ POD. GL was confirmed by a CT scan and endoscopic control, with contextual EDPC left in place.

### 1.5. Case 4

A case of intermediate GL occurred on the 6^th^ POD without clinical or biochemical worrisome features. A GST test performed on the 2^nd^ POD outlined a borderline situation, without radiological signs of GL. Quality of surgical drain became suggestive for GL on the 5^th^ POD, and GL was then confirmed by CT.

### 1.6. Case 5

Scenario 5: a male patient affected by tardive GL occurred on the 9^th^ POD. A large defect was identified at endoscopic control. After placement of EDPC, the clinical scenario required the placement of a nasojejunal tube for enteral nutrition.

### 1.7. Follow-up

Usually, the patient resumes an oral diet the day after the endoscopic procedure. A CT scan with gastrografin was performed at 1 month to evaluate the collection and the patency of the fistula. If the collection has not yet been reabsorbed, a stent exchange was performed according the new size and shape of the fistula, to promote granulation and to improve the internal drainage (Case 5); if the reabsorption was achieved, the stent removal was performed, and the resolution was confirmed by the endoscopic fistulography.

For 5 patients of our series, follow-up was more strict than usual and consisted of a clinical visit and biochemical test on the 7^th^, 14^th^, and 21^th^ day after discharge and a C -scan with gastrografin on the 30^th^ day from discharge if clinical visit and exams were normal.

In our cases, clinical, biochemical, and instrumental tests confirmed complete resolution of GL, with promotion of a pseudodiverticula and complete re-epithelialization of leak. The internal pig tail was endoscopically removed from the 30^th^ (Case 1–4) to 40^th^ POD (Case 5) in all cases. Clinical visit and biochemical tests were repeated at 3, 6, and 12 months after removal. All controls showed complete resolution of the complication.

## 2. Methods

From March 2014 to June 2019, 117 patients underwent laparoscopic sleeve gastrectomy (LSG) at our surgical department (Table 2). The surgical technique and team were always the same.

Protocol of management of patients affected by morbid obesity provides well defined procedures, including a psychological and nutritional preoperative supervision. According with SICOB guidelines [5], we propose LSG to patients with a body mass index (BMI) included between 40 and 45 or between 35 and 40 with diagnosed comorbidities (hypertension, hyperlipemia, diabetes, invalidant arthrosis, or back low pain) [5, 6, 8].

### 2.1. Operative Procedures

The standardized surgical technique begins with the placement of 4 ports and, after exploration of the sovramesocolic area, using an energy device (Ultracision® by Ethicon), and dissection of the greater curvature from the stomach paying attention to gastroepiploic vessels. A 36-French bougie is placed into the stomach to fashion the right dimension of sleeve gastrectomy, and the suture is performed by Echelon Flex™ ENDOPATH® Staplers (Ethicon). We placed one negative suction drain tube along the staple line, which is removed on the 5^th^ postoperative day (POD). A nasogastric tube is not needed. A blue dye test is usually performed before the ending intervention, to confirm tightness of the linear suture and exclude the presence of leakage. In addition, we used to perform a Indocyanine Green (ICG) test intraoperatively, to evaluate adequate blood perfusion of staple line, especially next to the critical angle of His [9].

### 2.2. Postoperative Management

About postoperative management [10], fasting is necessary for the 1^st^ POD, such as a biochemical test including C-reactive protein (CRP) and procalcitonin (PCT), repeated on the 3^rd^ and 5^th^ POD; a gastrografin swallow test (GST) is performed on the 2^nd^ POD; and a clear liquid diet is allowed on the 3^rd^ POD and soft aliments (for example, fruit mousse) on the 4^th^ POD, with negative GST. From the 5^th^ POD, an adequate diet is introduced in accord with the nutritional team; discharging is allowed on the 5^th^/6^th^ POD. Normally, a regular diet is recommended after 2 weeks from surgery if no complications occur. Regular follow-up is recommended for all patients and requires clinical and laboratory exams at 2 weeks from surgery; clinical and GST at 1 month; clinical and laboratory exams at 3 months; and clinical examination after 6, 12, 18, and 24 months [5, 6, 8].

### 2.3. Endoscopic Procedures

All patients with diagnosis of GL were referred to the endoscopy department of our hospital for GL treatment with placement of EDPC. After obtention of informed consent, patients underwent gastroscopy.

All procedures were performed with propofol sedation with patients in the supine position to better visualize the fistula and adopting the anti-Trendelemburg position to avoid, as much as possible, contrast reflux and inhalation during the procedure. Carbon dioxide insufflation (Olympus UCR, Olympus Europe, Hamburg, Germany) was used in all cases.

A therapeutic gastroscope (Olympus GIF-1T140, Olympus Europe, Hamburg, Germany) with a 3.7 mm working channel was used to easily deploy large stents if needed, with the help of a single-use, soft, fenestrated distal attachment (Olympus Europe, Hamburg, Germany) to improve the field of view, and to stabilize the scope tip during the maneuvers. Accurate endoscopic evaluation is mandatory to identify the fistula orifice, its shape, and location.

In case of failure, a duodenoscope (Olympus TJF-Q180 V, Olympus Europe, Hamburg, Germany) can be a viable option. The evaluation of the gastric remnant is furthermore crucial to exclude twist or stenosis conditioning the internal drainage performance.

After wall defect identification, the fistula cannulation was obtained with a 6Fr catheter with radiopaque markers (Cook Medical Europe, Limerick, Ireland) and a fluid was aspirated for microbiologic colture. A washing of the collection was performed with saline, and the opacification of the fistula was performed by gastrografin injection to visualize the fistulous tract and the size and shape of the collection. This step is of paramount importance to decide the correct length and diameter of the pig-tail stent and its number to accomplish an adequate drainage of the collection. After introduction of a 0.0035 angulated guidewire by the catheter, the appropriate stent (Advanix, Boston Scientific, Boston, Massachussets, USA) was deployed.

## 3. Discussion

The leaks reported in our series are seemingly unusual in frequency (4.3%-Table 2) and in timing, with many appeared after IV POD. This trend, despite being at the upper limits of normality reported in the literature [4, 10–12], can be explained considering the surgical learning curve of our department (that is accredited by Italian society of bariatric surgery from 2018).

Our bariatric department is not a high-volume center: in order to avoid unnecessary discomforts to our patients and an effective increase of regional healthcare-system expense, we prefer to discharge patients on 5^th^–6^th^ POD (high-volume bariatric center discharged patients on 2^nd^–4^th^ POD [5, 13]), consequently whit realimentation and relative safety about appearance of surgical complication.

In order to identify and treat this fearsome complication as soon as possible, our center adopted a discharge careful approach.

The work in [11, 14] confirms that the most important clinical signs in patients with GL are fever and tachycardia (others agree that tachycardia is the earliest, most important, and constant clinical finding, indicating the presence of GL; a tachycardia above 120 beat/min is a powerful indicator of leak and systemic compromise), which mandate the use of an abdominal CT, associated with an upper gastrointestinal series and/or gastroscopy.

Also, in our series, tachycardia and fever are the most important clinical factors in the diagnosis of GL post-LSG, and of course, the diagnostic management is evidence-medicine based.

Surgical reintervention (laparoscopic suture of GL or conversion of LSG in Roux-en-Y Gastric bypass- RYGB) could be understood as the best first-line treatment [15]. Even if surgical guidelines are not standardized yet, a lot of literature dates show that rate of GL can increase following repeated surgery (physiological perfusion of gastroesophageal junction is often missed in all the upper part of the gastric tube after LSG) [4]. Also considering that common surgical complications can occur and insist on a patient already undergone to a failed surgery [16], surgery should be considered more correctly as the last line of treatment and, however, in case of failure of less invasive procedures [17, 18]. Also, RYGB has similar complications to LSG and requires longer operative times, major hospitalization, and increasing costs. Overall leak rate-related mortality is low (0.6%) in RYGB; however, leak-associated mortality is significantly higher (14.7%–17%) [18, 19]. The results are similar in the SG population with an incidence of 1%–2.7% [20], overall leak-related mortality 0.14%, and leak-associated mortality 9%.

Available endoscopic approaches for after sleeve-gastrectomy leaks (PSGL) range from primary and secondary closure techniques by the use of endoluminal sewing devices, over-the-scope clips, fibrin glue, and diversion with self-expanding covered or partially covered metal stents, to EID techniques with the use of nasocystic drains or double-pigtail stents, endoscopic vacuum therapy, and septotomy with or without pneumatic dilation of the distal sleeve obstruction [17].

Because primary endoscopic closure is rarely feasible or successful for chronic leak and fistula management, endoscopic internal drainage (EID) [21] by means of a double-pigtail stent insertion is increasingly used as an effective approach for the management of leak and fistula following bariatric surgery. This new approach focuses on optimizing pressure gradients to allow internal drainage of the external collection with closure of the cavity by secondary intention, through granulation tissue formation and fibrosis.

This endoscopic approach has several potential advantages [22], including its minimally invasive nature, lack of influence of BMI, and the minimal inflammation produced by the procedure, which does not interfere with the healing process [23].

For subacute or chronic leaks with an organized walled-off collection, EDPC is effective both clinically and from a cost perspective analysis [24]. Whenever feasible, endoscopic exploration of the perigastric cavity can be performed to clean and remove necrotic infection and enhance drainage. Managing a downstream stenosis, twist, or kink within the sleeve that creates an unfavorable pressure gradient in this situation is crucial to enhance drainage and resolution.

Unfortunately, all the techniques described above have reported various degrees of both technical and clinical success and associated adverse events, generating a lack of consensus compounded by the exiguity of randomized trials that evaluate these different approaches.

A word of caution [25]: special situations including formation of gastropleural or gastrocutaneous fistulas require referral to a tertiary bariatric center with both endoscopic and surgical expertise. In these cases, a minimally invasive hybrid endoscopic and laparoscopic approach that disrupts the fistula and subsequent repair with a tailorable bioabsorbable plug matrix has been recently proposed [26, 27].

### 3.1. Limitations

There are several limitations in our study: (i) this study was conducted in a single low-volume center, for homogeneity of the EDPC technique; (ii) the study is retrospective and not prospective; and (iii) the number of cases is relatively low insufficient, and the results may be biased.

With these limitations in mind, our data show that EDPC may be effective as first-line treatment in case of GL after LSG. Of course, our results can be considered as preliminary data about GL endoscopic treatment.

## 4. Conclusions

Cases of PSGL and fistulas will likely continue to rise despite improvements in techniques, given the rising number of bariatric procedures performed. Surgeons and endoscopists should refine their collaboration, recognizing the underlying causes leading to leak formation and perpetuity, to individualize endoscopic therapy [19].

EDPC maintains the characteristic of safety and efficacy, and we consider it as first line of treatment in case of GL after bariatric surgery. A relevant vantage of EDPC remains the possibility of GL early management: this can avoid the worst complication such as necrosis of the gastroesophageal junction, septic state, or hemorrhage complications. EDPC acts like a foreign body and promotes the physiological re-epithelialization of the leak, just like common surgical abdominal drains.

EDPC allows an early treatment of GL after LSG and is associated with shorter hospital stay and reduction of global costs. Also, our series never needed an Intensive Care Unit (ICU) because of the minimally invasive approach of EDPC.

Patients' perspective is very important to evaluate the usefulness of EDPC: the possibility of early discharge if compared to other known approaches makes it very appreciated from most patients.

Finally, compared to RYGB, EDPC does not contraindicate further endoscopic evaluation of the Upper GI tract (no modifications of normal anatomy).

## Figures and Tables

**Table 1:** Summary of the complicated group (GL group) after LSG.

Name (sex)	Birthday patient	Date of sleeve gastrectomy	Date of gastric leak	Placement of EDPC
S. A. (F)	04/07/1962	12/09/2016	II POD	IV POD
M. J. (F)	17/06/1988	13/10/2016	VI POD	VII POD
M. F. C. (F)	01/10/1961	05/03/2018	VIII POD	VIII POD
D. L. L. (F)	17/06/1969	21/05/2018	VI POD	VIII POD
S. A (M)	30/07/1967	24/06/2019	IX GPO	XIII POD

**Table 2:** Demographic, clinical, surgical, and pathologic details of the study population.

Parameter	Total, *n* (%)
*AGE (years), mean*	117 (100%)
≥50	45 ± 9
<50	73 (62)

*Gender*	44 (38)
Male	39 (33)
Female	78 (67)

*BMI (kg/m* ^*2*^ *), mean*	41.6 ± 4
≥41.6	56 (48)
<41.6	61 (52)

*Comorbidities*	
Type 2 DM	19 (17)
Hypertension	34 (29)
Dyslipidemia	44 (37)
Obstructive sleep apnea	46 (39)

Perioperative blood trasfusion	1 (1)
Conversion	0 (0)
Prolonged time surgery (>2 h)	4 (3)
Gastric leak	5 (4)
Prolonged hospital los (>4 days)	9 (8)
Readmission	0 (0)
Mortality	0 (0)

## Data Availability

The data used to support the findings of the study are available from the corresponding author upon request.

## References

[1] Welboum R., Hollyman M., Robin K. (2019). Bariatric surgery Worldwide: baseline demographic description and one-year outcomes from the Fourth IFSO Global Registry Report 2018. *Obesity Surgery*.

[2] Ogden C. L., Carroll M. D., Cynthia L. O. (2015). Prevalence of obesity among adults and youth: United States, 2011–2014. *National Center for Health Statistics Data Brief*.

[3] Rosenthal R. J., Diaz A. A., Dag A. (2012). International sleeve Gastrectomy expert panel consensus statement: best practice guidelines based on experience of >12,000 cases. *Surgery for Obesity and Related Diseases*.

[4] Walsh C., Karmali S. (2015). Endoscopic management of bariatric complications: a review and update. *World Journal of Gastrointestinal Endoscopy*.

[5] SICOB Linee Guida di Chirurgia dell’Obesità (2016). *Società Italiana di Chirurgia dell’Obesità e della Malattie Metaboliche*.

[6] SAGES Guidelines Committee (2009). SAGES guideline for clinical application of laparoscopic bariatric surgery. *Surgery for Obesity and Related Diseases*.

[7] Corona M., Zini C., Carlo C. (2012). Minimally invasive treatment of gastric leak after sleeve gastrectomy. *Radiological Medicine*.

[8] Sauerland S., Angrisani L., Belachew M. (2005). Obesity surgery: evidence-based guidelines of the European association for endoscopic surgery (EAES). *Surgical Endoscopy*.

[9] Di Furia M., Romano L., Antonio G. (2019). Indocyanine green fluorescent angiography during laparoscopic sleeve gastrectomy: preliminary results. *Obesity Surgery*.

[10] Deitel M., Gagner M., Erickson A. L., Crosby R. D. (2011). Third International Summit: current status of sleeve gastrectomy. *Surgery for Obesity and Related Diseases*.

[11] Burgos A. M., Braghetto I., Csendes A. (2009). Gastric leak after laparoscopic-sleeve gastrectomy for obesity. *Obesity Surgery*.

[12] Sarela A. I., Dexter S. P. L., O’Kane M., Menon A., McMahon M. J. (2012). Long-term follow-up after laparoscopic sleeve gastrectomy: 8-9-year results. *Surgery for Obesity and Related Diseases*.

[13] Giuliani A., Romano L., Marchese M. (2019). Gastric leak after laparoscopic sleeve gastrectomy: management with endoscopic double pigtail drainage. A systematic review. *Surgery for Obesity and Related Diseases*.

[14] Nedelcu A. M., Skalli M., Deneve E., Fabre J. M., Nocca D. (2013). Surgical management of chronic fistula after sleeve gastrectomyﬁstula after sleeve gastrectomy. *Surgery for Obesity and Related Diseases*.

[15] Aurora A. R., Khaitan L., Saber A. A. (2012). Sleeve gastrectomy and the risk of leak: a systematic analysis of 4,888 patients. *Surgical Endoscopy*.

[16] Khoursheed M., Al-Bader I., Mouzannar A. (2016). Postoperative bleeding and leakage after sleeve gastrectomy: a single-center experience. *Obesity Surgery*.

[17] Sakran N., Goitein D., Raziel A. (2012). Gastric leaks after sleeve gastrectomy: a multicenter experience with 2,834 patients. *Surgical Endoscopy*.

[18] Vargas E. J., Abu Dayyeh B. K. (2018). Keep calm under pressure: a paradigm shift in managing postsurgical leaks. *Gastrointestinal Endoscopy*.

[19] Fernandez A. Z., DeMaria E. J., Tichansky D. S (2004). Experience with over 3,000 open and laparoscopic bariatric procedures: multivariate analysis of factors related to leak and resultant mortality. *Surgical Endoscopy*.

[20] Lee S., Carmody B., Wolfe L. (2007). Effect of location and speed of diagnosis on anastomotic leak outcomes in 3828 gastric bypass cases. *Journal of Gastrointestinal Surgery*.

[21] Donatelli G., Dumont J.-L., Cereatti F. (2016). Endoscopic internal drainage as first-line treatment for fistula following gastrointestinal surgery: a case series. *Endoscopy International Open*.

[22] Donatelli G., Fuks D., Cereatti F. (2018). Endoscopic transmural management of abdominal fluid collection following gastrointestinal, bariatric, and hepato-bilio-pancreatic surgery. *Surgical Endoscopy*.

[23] Romano L., Mattei A., Colozzi S. (2020). Laparoscopic sleeve gastrectomy: a role of inflammatory markers in the early detection of gastric leak. *Journal of Minimal Access Surgery*.

[24] Bemelman W. A., Baron T. H. (2018). Endoscopic management of transmural defects, including leaks, perforations, and fistulae. *Gastroenterology*.

[25] Ghanem O. M., Abu Dayyeh B. K., Kellogg T. A. (2017). Management of gastropleural fistula after revisional bariatric surgery: a hybrid laparoendoscopic approach. *Obesity Surgery*.

[26] Debs T., Petrucciani N., Kassir R. (2017). Migration of an endoscopic double pigtail drain into the abdominal wall placed as a treatment of a fistula post revisional bariatric surgery. *Obesity Surgery*.

[27] Baltasar A., Serra C., Pérez N., Bou R., Bengochea M., Ferri L. (2005). Laparoscopic sleeve gastrectomy: a multi-purpose bariatric operation. *Obesity Surgery*.

